# Acceptability and Usefulness of Telenursing from Nurses’ Perspectives: A Descriptive Pilot Study

**DOI:** 10.3390/healthcare14162491

**Published:** 2026-08-11

**Authors:** Fabrizio Petrone, Simona Sgromo, Sara Dionisi, Alessandro Spano, Emanuele Di Simone, Gloria Liquori, Noemi Giannetta, Priscilla Molinaro, Marco Di Muzio, Nicolò Panattoni, Lorenzo Pietropaolo, Aurora De Leo

**Affiliations:** 1 Nursing Research Unit IFO, IRCCS Regina Elena National Cancer Institute, 00144 Rome, Italy; fabrizio.petrone@ifo.it (F.P.); simona.sgromo@ifo.it (S.S.); alessandro.spano@ifo.it (A.S.); aurora.deleo@ifo.it (A.D.L.); 2 Department of Biomedicine and Prevention, University of Rome Tor Vergata, 00133 Rome, Italy; 3 Nursing, Technical, Rehabilitation Department, DaTeR Azienda Unità Sanitaria Locale di Bologna, 40124 Bologna, Italy; srdionisi@gmail.com; 4 Department of Medical, Movement and Wellbeing Sciences, Parthenope University of Naples, 80133 Napoli, Italy; emanuele.disimone@uniparthenope.it; 5 Unit of First Aid, Emergency Department, San Filippo Neri Hospital, ASL Roma 1, 00193 Rome, Italy; gloria.liquori@gmail.com; 6 Departmental Faculty of Medicine, Saint Camillus International University of Health and Medical Sciences (UniCamillus), 00131 Rome, Italy; noemi.giannetta@unicamillus.org; 7 Department of Wellbeing, Health and Environmental Sustainability, Sapienza University of Rome, 00185 Rome, Italymarco.dimuzio@uniroma1.it (M.D.M.); 8 Faculty of Pharmacy and Medicine, Sapienza University of Rome, 00185 Rome, Italy; pietropaolo.2171684@studenti.uniroma1.it

**Keywords:** attitudes, barriers, knowledge, nurse, pilot study, predictors, questionnaire, telenursing

## Abstract

**Highlights:**

**What are the main findings?**

- Nurses’ knowledge and skills on telenursing, its impact on communication, the nurse-patient relationship, perceived benefits, professional outcomes and organisational support are key factors in the spread of these tools.
- Although the analysis suggested good internal consistency (α = 0.806), a further validation study with a larger sample size is required to assess the psychometric properties of the tool.

**What are the implications of the main findings?**

- The future assessment of the psychometric properties of the questionnaire to measure nurses’ attitudes and barriers towards telenursing could be a useful organisational tool to provide valuable insights into nurses’ perceptions and perspectives regarding telenursing interventions.

**Abstract:**

**Background**: Nurses’ perspectives on telenursing are essential to improving its use by patients and nurses. **Objective**: This descriptive pilot study aims to assess the acceptability and usefulness of telenursing among nurses through a questionnaire developed by the authors. Additionally, the internal consistency of the instrument was assessed. **Methods**: A cross-sectional descriptive pilot study was conducted using an online survey and a 43-item questionnaire in October 2024. Convenience sampling was used to enrol interested nurses employed in any setting. Inferential statistical tests were conducted to assess the associations between the variables. Additionally, the internal consistency of the questionnaire was tested using Cronbach’s alpha. The study was conducted in accordance with the Strengthening the Reporting of Observational Studies in Epidemiology (STROBE) guidelines. Consent was obtained from the involved nurses and the relevant Ethics Committee. **Results**: In total, 53 Italian nurses were included. A total of 94.4% of them would use telenursing in clinical practice and would be interested in attending educational programmes on telenursing (86.8%). The analysis suggested that nurses’ positive attitudes towards telenursing are predicted by their gender (being female), knowledge, strategic management support, and the time-saving potential of telenursing. A Cronbach’s α of 0.86 suggested a good internal reliability of the questionnaire. **Conclusions**: The study findings suggest positive nurses’ acceptability and utility for telenursing, positively predicted by gender, knowledge, potential time-saving benefits of telehealth nursing, and organisational support. A positive attitude towards telenursing could encourage its use among nurses and patients. These preliminary findings should be confirmed in a future validation study.

## 1. Introduction

Telenursing is a relatively new nursing model that uses information and communication technologies to improve the quality and continuity of nursing care and patient satisfaction [1]. According to the International Council of Nurses [1], it enhances the effectiveness, efficiency, and appropriateness of traditional nursing care. In addition, it promotes interaction between nurses, healthcare professionals, and patients, overcoming barriers such as distance and time [2]. 

Despite the increasing use of telenursing, particularly after the recent SARS-CoV-2 pandemic, its adoption and use are heterogeneous worldwide. Recent studies suggest that nurses’ perceptions, acceptability, usefulness, and preconceptions about telenursing may influence its use and uptake among professionals and patients [3], suggesting the need to consider their barriers and preferences for promoting its use. International studies suggest that the implementation of these tools depends on both the characteristics of the providers and those of the community [4,5,6]. In particular, poor ease of use and lack of training have been identified as barriers to the use and adoption of these tools [5], contrary to a positive attitude among nurses [6].

To the authors’ knowledge, there are no validated tools to assess nurses’ views on the acceptability and usefulness of telenursing.

This descriptive pilot study aims to assess the acceptability and usefulness of telenursing among nurses. The instrument’s internal consistency was also assessed, along with factors associated with the use of telenursing in the sample. A subsequent study will be conducted to validate the questionnaire based on the preliminary results of this study. The questionnaire was developed by the authors, based on a preliminary literature review [4,5,6,7], the Knowledge, Attitudes, Behaviours (KAB) framework [8], and a previous descriptive study conducted by the research team on the topic [9].

## 2. Materials and Methods

A descriptive pilot study was conducted using an online survey to assess the acceptability and usefulness of telenursing among nurses. Furthermore, the internal consistency of the questionnaire was assessed. The questionnaire used was a self-administered instrument developed ad hoc by the authors (Supplementary File S1). In line with guidelines for testing the validity and reliability of questionnaires, the pilot phase data collection aimed for a sample of ≥30 respondents [10]. Strengthening the Reporting of Observational Studies in Epidemiology (STROBE) Guidelines [11] were used to improve the reporting. The study was conceived and developed within a research team from a National Cancer Center. The team consisted primarily of nurses with PhDs and experience in quantitative and qualitative research and study validation.

### 2.1. Recruitment and Sampling

In accordance with Beaton’s guidelines [12], convenience sampling was used to achieve the aims of the study. The online survey was shared among nurses employed in Italy via email, using non-probability, convenience, and voluntary sampling. Nurses’ participation was free and voluntary, shared between interested nurses, and subject to signing an informed consent form in electronic format. Due to the pilot nature of the study and following the guidelines for questionnaire validation [10], this descriptive pilot study involved a sample of ≥30 respondents, and a formal sample size calculation was not performed.

### 2.2. Inclusion and Exclusion Criteria

The convenience sampling strategy was informed by the following criteria:

Inclusion criteria: Registered nurses employed in Italy, aged ≥18 years old; nurses working in any clinical, organisational, educational, and research contexts in Italy; Italian mother-tongue nurses willing to comply with the study procedures for data collection, analysis, and dissemination. 

Exclusion criteria: undergraduate nursing students, nursing staff and other healthcare professionals, retired nurses, and nurses unable to speak Italian and comply with the study procedures.

### 2.3. Tools

In a previous descriptive study conducted by the research team in August 2022, 323 nurses were interviewed about their views on telenursing using an early version of a specially developed instrument [9]. Questionnaire items were developed by the same research team, following an extensive literature review [4,5,6,7] and the KAB framework theory [8]. The research team consisted of three PhD nurses, one MSN nurse, one MSNc nursing student, two nurse practitioners, and a professor of nursing who was an expert in statistics and supervised the study. The PhD nurses and the professor of nursing were experienced in study validation [8,9,13]. The guidelines for questionnaire validation were followed to ensure the validity of the data collected [10] to assess the acceptability and usefulness of telenursing from the nurses’ perspective. Following the results and the critical issues of the preliminary descriptive study [9], a review of the items was undertaken by the research team. The instrument used in this descriptive pilot study was the revised 43-item questionnaire of the original tool [9] (Supplementary File S1). The instrument included four main sections to describe the socio-demographic (four items related to gender, nationality, region, age) and professional (seven items related to qualifications, experience and clinical context) characteristics of the sample, as well as knowledge and attitudes regarding the use of telenursing (sixteen items related to use of the Internet and telenursing in clinical contexts, self-assessed and measurable nurses’ knowledge and opinions about telenursing, its impact on time saving, professional responsibility, skills, guidelines, preferred tools, fears, confidence, interest, perceived satisfaction and the need for training), and potential resistance to its use (sixteen items related to cultural barriers, lack of operational support and management involvement, lack of resources, clinical use in acute and chronic care, therapeutic education and palliative care, the complexity of the tools and patients’ barriers). Knowledge, attitudes, and barriers were described by single- or multiple-choice items using five-point Likert scales (Strongly agree, Agree, Neutral, Disagree, and Strongly Disagree). Some items asked respondents to self-assess their knowledge, attitudes, and barriers to the topic (e.g., “My knowledge of using telenursing is good”), while others explored their point of view based on national and international research (“Nurses using telenursing have the same professional, ethical, and legal responsibilities as in traditional care to provide safe, competent, compassionate and ethical care”). The items were both mandatory and closed. Enrolment in the study and the completion of the questionnaire were preceded by a brief introductory section describing the purpose, objectives and methods of data collection, processing and analysis. 

### 2.4. Data Collection Procedures

The self-administered questionnaire was shared with interested nurses via email through a Google Form tool in October 2024. The survey link was shared via company emails. Participation was free, voluntary, and without any financial incentive to force nurses to participate. Before the questionnaire, an information section explained to participants the purposes, objectives, data collection and analysis procedures, and protection of their privacy. The enrollment of the interested nurses was defined by the signature of a mandatory digital informed consent, collected before completing the questionnaire. Participant information was collected anonymously and stored on the study principal investigator’s personal computer, which was password-protected and accessible only to the research team. Participant data were collected in an Excel spreadsheet for subsequent analysis. To ensure completeness of the information, all questionnaire items were mandatory. Therefore, incomplete or missing information was not collected. Given the pilot nature of the study and following the guidelines for questionnaire validation [10], this descriptive pilot study used a convenience sampling strategy of ≥30 eligible nurses.

### 2.5. Statistical Analysis

Data collection, analysis, and summary were consistent with the study aims. Absolute frequencies and percentages (%) were used for categorical variables. For continuous, normally distributed variables, means ± standard deviations (SD) or medians and ranges were used. *p*-value < 0.05 was considered statistically significant. The internal consistency of the questionnaire was assessed through Cronbach’s alpha. Good internal consistency of the instrument was considered for Cronbach’s alpha values ≥ 0.70. Linear Regression Analysis was used to identify factors associated with the use and acceptability of telenursing among nurses. 

The evaluation of the psychometric properties of the instrument will be tested in a future validation study [14,15].

Statistical Package for Social Science v. 29.0 software (SPSS, Chicago, IL, USA) was used for data analysis.

### 2.6. Ethical Considerations

This descriptive pilot study was approved by the Lazio 5 independent Regional Ethics Committee (No. 227/IRE/24) on 25 September 2024 and was conducted in accordance with the ethical principles of the revised Declaration of Helsinki [16]. Nurses participated on a free and voluntary basis. No compensation was provided. Participants gave formal informed consent to be included in the study, and no withdrawals were recorded. Information about the purpose, aims, and methods of data collection, processing, analysis, and dissemination was provided to all interested nurses in a short introductory section (Supplementary File S1). The email address of the principal investigator was provided at the end of this section in case participants had any further questions. The aggregated and pseudonymised data were collected, processed, managed and archived electronically, solely for the aims of the study and in accordance with data minimisation principles and respect for privacy. The results are presented in aggregated form and do not allow the identification of the opinions of the participants.

The study was neither conducted nor reported using any artificial intelligence-generated content (AIGC), such as ChatGPT (OpenAI, San Francisco, CA, USA) and other tools based on large language models (LLMs).

## 3. Results

A total of 53 nurses were involved in this descriptive pilot study. No withdrawal or missing data were observed.

### 3.1. Characteristics of the Sample

100% of the nurses surveyed were Italian and mainly employed in the centre of the country. In total, 56.5% (N = 30) of the sample were aged between 20 and 40 years, and 54.7% (N = 29) had been employed for less than 15 years. A total of 75.4% (N = 40) of the nurses involved were postgraduate. In terms of clinical context, 67% (N = 36) of the participants were employed in public hospitals. Table 1 shows the main socio-demographic and professional characteristics of the sample.

### 3.2. Nurses’ Knowledge and Attitudes

The academic background of the majority of the sample (84.9%, N = 45) did not include the use of telenursing, although 28.3% (N = 15) of them are sure that they have a good level of knowledge about telenursing. A total of 94.4% of nurses (N= 50) would have no objection to the use of telenursing in clinical practice and would be interested in attending educational programmes on the topic (86.8%, N = 46) (Table 2). 

Most of the sample considered these tools as a means to save time and increase job satisfaction, would not be afraid to use them in clinical practice, and agreed that remote communication based on careful nonverbal language and the use of open-ended questions was useful in facilitating remote patient-nurse interactions. In total, 49 nurses (92.5%) agreed that telenursing and traditional face-to-face clinical practice had the same professional responsibilities, in contrast to the 4 (7.5%) nurses who were not sure. From the perspective of the nurses involved, technological issues, privacy, and lack of professional competence could be the main problems associated with the use of telenursing. Finally, 45 nurses (84.9%) were not aware of any specific guidelines for the use of telenursing and felt that video calls, apps, and platforms (83%, N = 29) would be the most appropriate tools for these activities. Table 3 shows the nurses’ knowledge and attitudes towards telenursing.

### 3.3. Nurses’ and Patients’ Barriers

Most nurses felt that telenursing could improve or not affect the patient-nurse relationship (88.7%, N = 47), team communication, and patient safety issues (83%, N = 44). The main barriers for nurses to use telenursing were a lack of support and involvement from their general management, a lack of familiarity with digital resources, cultural issues such as wrong beliefs, training and updating, a lack of time, available equipment, and dedicated staff. In this regard, 88.7% (N= 47) of participants suggested that sharing and involving nurses in the strategic goals of the organisation could promote satisfaction and use of telenursing. Table 4 shows nurses’ barriers.

Most nurses perceived a lack of digital literacy and prejudice as potential barriers, which they attributed to patients, as shown in Table 5. No nurse (0%, N = 0) would be opposed to educational and informative nursing interventions aimed at patients and caregivers to reduce these barriers. The main applications and areas where telenursing could be more effective were also assessed by the tool, as shown in Table 5. Overall, most of the sample considered telenursing effective, particularly in chronic disease management, followed by patient and caregiver education, postoperative monitoring of vital signs and symptoms, and palliative care. The least appropriate setting for telenursing was perceived to be acute care (58.4%, N = 31).

Linear regression analysis (Table 6) suggested statistically significant associations (*p* < 0.05) in the following areas: female gender, improved communication and knowledge of telenursing guidelines; the perceived level of knowledge on telenursing and time savings, communication, professional liability and usability; time savings on using telenursing and organisational barriers; communication and patient barriers towards telenursing.

A Cronbach’s alpha of α = 0.86 suggested a good internal consistency of the questionnaire.

Results have been reported in line with the STROBE checklist to ensure completeness and transparency (Supplementary File S2).

## 4. Discussion

The use of telenursing has recently increased among nurses in a broad range of settings, with a positive impact on healthcare outcomes [17,18]. Knowledge of nurses’ perspectives on telenursing may therefore be crucial in promoting its use between nurses and patients [3].

This descriptive pilot study was conducted to assess the acceptability and usefulness of telenursing from nurses’ perspectives. Moreover, the internal consistency of the 43-item questionnaire was assessed. The questionnaire was developed based on KAB theory [8], a previous extensive literature review on the topic [4,5,6,7], and a preliminary observational study [9] conducted by the research team. Following this descriptive pilot study, a further cross-sectional study will be conducted to test the psychometric properties of the tool on a larger sample [14,15]. The questionnaire is intended to be a supportive self-assessment tool for nurses’ perspectives on the phenomenon. It could also be an effective tool for implementing targeted interventions and strategies to promote its use among healthcare professionals and patients, providing interesting and useful information to policymakers to improve nurse training and organisational support.

In line with a recent qualitative study [19], our findings suggested that nurses’ knowledge and skills in using telenursing, as well as its potential to improve the quality of care, communication, and nurse–patient relationships, could be strategic factors in promoting its use. Furthermore, nurses involved in the study considered the time-saving characteristics of telenursing and organisational support to be key elements that would promote its use. Cronbach’s α = 0.806 suggested a good reliability of the tool, which should be tested on a larger sample. 

The results of the linear regression analysis allowed the authors to highlight some interesting aspects of the sample involved. According to recent studies [20,21], the academic training of the 53 nurses involved in the study did not include the use of these tools in clinical practice (84.9%, N = 45). Despite the lack of academic training, most of the sample (69.8%, N = 37) self-reported a good awareness of telenursing. This apparent discrepancy could be explained by the fact that 75.5% (N = 40) of the respondents had further training, which could be used to study the subject in more depth. In addition, to overcome the bias introduced by respondents’ self-assessment of the areas under investigation, some questions assessed knowledge of formal recommendations for the use of telenursing. In this regard, greater knowledge of telenursing was also associated with nurses’ awareness of the professional responsibilities associated with this approach (*p* < 0.001), including those related to technological issues, privacy, and the skills required (*p* < 0.006). The professional mentoring they received on the job could also help to bridge the gap in basic telenursing training. Among the nurses interviewed, 84.9% (N = 45) had not received academic training on the use of telenursing interventions, and 64.2% (N = 34) did not use these tools in their work. Future research should clarify the level and types of telenursing use in the sample to avoid drawing generalisations from these gaps.

Finally, in line with the findings of the previous observational study [9] and recent studies [22], 86.8% (N = 46) of the participants would be in favour of attending telenursing training courses.

Respondents’ positive attitudes towards telenursing were supported by a marginal subset of nurses (6%, N = 2) who perceived telenursing as potentially compromising the quality of traditional nursing care. However, cross-cutting and multi-factorial strategies need to be implemented to overcome nurses’ barriers to adopting this new approach and to promote its use by patients [19]. In line with KAB theory [8], nurses’ greater knowledge of telenursing is significantly associated with their intention to use it. In this regard, according to recent studies [23], telenursing is perceived by the sample as a simple and useful tool to save time, improve communication and efficiency, and optimise work (*p* < 0.001). Furthermore, greater knowledge of telenursing is associated with a greater willingness to make the best use of these tools in clinical practice (*p* < 0.000), compliance with local regulations, professional accountability, and improved relationships with patients (*p* < 0.006). In line with recent studies [24], telenursing is seen by the sample as a means to improve communication within the care team and job satisfaction. The statistically significant association between female gender and effective remote communication (*p* < 0.023) could be an interesting area for future research. The sample also considers effective communication to be essential for the implementation of more effective educational strategies, as well as for overcoming patient reluctance to use these tools (*p* < 0.001). 

Recent studies suggest that, in this regard, the importance of the supportive effect and the satisfaction of the caller [25,26] is an area in which gender should play a crucial role. In line with the previous observational study [9], and contrary to recent literature [27], telephone support was considered the least appropriate tool for telenursing by the sample. Similar to previous findings [9], video calls were considered the most appropriate tool for telenursing. Furthermore, recent studies suggest that telenursing can support patients with symptom management, surgical care, and chemotherapy, particularly in cancer care [28,29,30,31,32,33]. Consistent with recent studies [34,35], the lack of operational support from management and the lack of knowledge (*p* < 0.001) are perceived as barriers to the use of these tools by the nurses involved in this descriptive pilot study. Organisational support could therefore play a strategic role in disseminating these tools between nurses and patients. Similarly, the nurses involved identified their involvement in organisational goals related to innovation as a strategic element to increase the use of these tools and professional satisfaction. In line with the international literature [36], our findings suggest that healthcare professionals should be considered as active participants in innovation change. Consistent with recent studies, nurses’ lack of confidence in telenursing tools, time commitment, and low digital literacy were the main barriers for the nurses involved in the study [37,38]. Therefore, future studies should explore and evaluate the appropriate use of telenursing at this time, taking into account the authors’ recommendations, nurses’ low confidence in these tools, time-related issues, and the low literacy level of the nurses involved in the study, as described in the extensive literature on the phenomenon [23,37,38]. In this regard, according to the authors, this descriptive pilot study aims to be a starting point for larger and more rigorous studies on the topic to fill specific gaps in this nursing approach.

Despite the barriers attributed to patients, nurses felt that patients could be generally supportive of telenursing, highlighting that only 6% (N = 2) of them could be clearly reluctant to use it. In line with previous studies [9,39], the complexity of the tools used and low digital literacy were also the main barriers attributed to patients. In this context, low digital literacy, especially among older patients, could be an important barrier to overcome in a global community with an increasing average age and consequently growing health needs [40]. Furthermore, according to our findings, and given the special relationship between nurses and patients, telenursing was seen by the sample as an effective tool for delivering educational programmes to patients and caregivers (*p* < 0.001), while at the same time reducing barriers to its use (90,6%, N = 48). 

Finally, this pilot study aims to be a starting point for larger studies on the topic, with a more rigorous methodology.

### 4.1. Limitations

The authors are aware of the limitations of the study. The first is the small sample size, which places significant limits on the generalizability of the results obtained. However, according to Beaton’s guidelines [12], this initial descriptive pilot study is a necessary stage in a future validation study to test the psychometric properties of the questionnaire on a larger sample. In total, 64.2% (34) of the sample did not use these tools in their clinical practice, and the questionnaire did not delve into the telenursing tools used by the remaining sample. Furthermore, this study is intended as a pilot study for the future development of a self-assessment tool to assess the acceptability and usefulness of telenursing among nurses. Several items required the nurse to self-assess the aspect under investigation. However, many items were developed based on national and international recommendations and guidelines for the use of telenursing. Many of the questions were structured on the assumption that respondents were using telenursing. Questions should likely include elements to assess which services should be implemented to facilitate the use of telenursing in the future. Future cross-sectional and qualitative studies among nurses could provide interesting information on this topic. In addition, an online survey does not ensure that only nurses were involved in the project, nor that each nurse responded to the web survey once. However, before analysis, the authors ensured that all email addresses used to access the survey were different.

### 4.2. Recommendations for Further Research and Practice

Among the nurses interviewed, 84.9% (N = 45) had not received academic training on the use of telenursing interventions, and 64.2% (N = 34) did not use these tools in their work. Future research should clarify the meaning, level, and types of telenursing use from the remaining nurses in the sample to avoid drawing generalisations from these gaps. In this regard, qualitative research could provide a useful perspective on this topic and support the authors in improving future validation of the questionnaire. Taking these preliminary findings into account, future studies should explore and evaluate the appropriate use of telenursing at this time, nurses’ confidence in these tools, time-related issues, the perceived lack of organisational support, and the low literacy level of the nurses involved in the study, as described in the international literature on the topic [23,37,38].

Although the analysis suggested good internal consistency (α = 0.806), a further validation study with a larger sample size is required to assess the psychometric properties of the tool. The statistically significant association between female gender and effective remote communication (*p* < 0.023) may be an interesting area for future research. The positive interest of the sample in telenursing should be further explored in future studies and supported by increased education and training of students and nurses. Knowledge of the factors that predict telenursing use among nurses could be particularly useful for cancer nurses when implementing remote interventions for cancer patients. Greater confidence among nurses in telenursing interventions and remote tailoring programmes could improve access to care for this vulnerable group, as well as enhance its safety and quality. The results of this preliminary study may particularly support the implementation of a randomised clinical trial that is currently underway at an Italian cancer centre [41]. Finally, the research team is currently conducting an in-depth qualitative study on nurses’ perceived risks at a National Cancer Center related to telenursing interventions and on safety considerations for nurses providing remote care. These aspects would also benefit from further investigation through longitudinal quantitative studies.

## 5. Conclusions

This descriptive pilot study produced some interesting findings concerning the significance of nurses’ knowledge and skills, acceptability and usefulness in telenursing, as well as its effect on communication, the nurse–patient relationship, perceived advantages, professional outcomes, and organisational endorsement. These preliminary findings could provide interesting and useful information to policymakers to improve the implementation of telenursing interventions. Specifically, they highlight the importance of improving nurses’ digital skills, digital and technological infrastructure, and organisational support strategies to spread the use of these tools among nurses and patients. Future studies should explore the lack of training for nursing students, their digital literacy, their interest in updating patients and nurses, the relevance of telenursing for therapeutic interventions, and nurses’ positive attitudes. Furthermore, this descriptive pilot study provided valuable insights into the future validation of the questionnaire on nurses’ use of telenursing. Future research is essential to ensure that telenursing improves the quality, access, and effectiveness of care and does not create additional barriers for people with fewer resources and greater healthcare needs [19,42]. Finally, these preliminary findings could offer interesting insights for consolidating further future research on human resource planning and development through ongoing nurse training on the use of telenursing in clinical practice.

## Figures and Tables

**Table 1:** Nurses’ socio-demographic and professional characteristics.

Socio-Demographic and Professional Characteristics	% (N)
**Age**	
20–30	35.8 (19)
31–40	20.7 (11)
41–50	22.6 (12)
51–60	20.7 (11)
**Gender**	
Male	26.4 (14)
Female	73 (39)
**Education**	
University Degree in Nursing	75.5 (40)
Non-University Qualification (Regional Course)	24.5 (13)
**Postgraduate Training**	
None	24.5 (13)
Master	62.2 (33)
PhD	13.2 (7)
**Year of Employment**	
0–2	15.1 (8)
3–5	5.7 (3)
6–10	24.5 (13)
11–20	30.2 (16)
>20	24.6 (13)
**Clinical Context**	
Medicine	7.5 (4)
Surgery	32.1 (17)
Pediatric Care	7.5 (4)
Critical Care	11.3 (6)
Management	5.7 (3)
Training and Research	20.8 (11)
Other	15.1 (8)

**Table 2:** Nurses’ education to telenursing.

Nurses’ Education	% (N)
**My knowledge on the use of telenursing is good**	
Strongly agree/Agree	69.9 (37)
Neutral	18.9 (10)
Disagree/Strongly disagree	11.3 (6)
**Did your academic training include the use of telenursing interventions?**	
No	84.9 (45)
Yes	7.5 (4)
e-Health only	5.7 (3)
Telenursing only	1.9 (1)
**Do you use telenursing at work?**	
No	64.2 (34)
Yes	35.8 (19)
**Are you interested in training in the use of telenursing tools in a clinical setting?**	
No	13.2 (7)
Yes	86.8 (46)

**Table 3:** Nurses’ knowledge and attitudes to telenursing.

Nurses’ Knowledge and Attitudes	% (N)
**I use the Internet more for work than leisure.**	
Strongly agree/Agree	52.8 (28)
Neutral	17 (9)
Disagree/Strongly disagree	30.2 (16)
**Remote nursing care, in addition to traditional care, enables nurses to provide care**	
Higher quality compared to traditional care	45.3 (24)
Same quality as traditional care	35.8 (19)
Lower quality compared to traditional care	7.5 (4)
I don’t know	11.3 (6)
**Telenursing saves time in clinical practice**	
Strongly agree/Agree	75.5 (40)
Neutral	20.8 (11)
Disagree/Strongly disagree	3.8 (2)
**Remote nurse-patient interactions are facilitated by open-ended questions and attention to non-verbal language, emotional and behavioural aspects**	
Strongly agree/Agree	88.7 (47)
Neutral	9.4 (5)
Disagree/Strongly disagree	1.9 (1)
**Telenursing has the same professional, ethical and legal responsibilities as traditional care to provide safe, competent, compassionate and ethical care**	
Strongly agree/Agree	92.5 (49)
Neutral	7.5 (4)
Disagree/Strongly disagree	0 (0)
**Professional liability issues in the use of telenursing include technology failure, confidentiality of health information, consistency of delivery methods, and telenursing-related skills**	
Strongly agree/Agree	79.3 (42)
Neutral	13.2 (7)
Disagree/Strongly disagree	7.5 (4)
**The tool I prefer to use and consider most suitable for telenursing is**	
e-mail	3.8 (2)
Messaging	7.5 (4)
Phone calls	1.9 (1)
Video calls	50.9 (27)
Applications an platforms	35.8 (19)
Other	0 (0)
**Guidelines for telenursing interventions and their use to support nurses are currently not specifically recommended**	
Strongly agree/Agree	59.5 (31)
Neutral	26.4 (14)
Disagree/Strongly disagree	15.1 (8)
**I would feel comfortable providing telenursing interventions**	
Strongly agree/Agree	64.2 (34)
Neutral	30.2 (16)
Disagree/Strongly disagree	5.7 (3)
**I would be concerned about providing telenursing interventions**	
Strongly agree/Agree	9.4 (5)
Neutral	15.1 (8)
Disagree/Strongly disagree	28.3 (15)
**Telenursing enhances professional development and job satisfaction**	
Strongly agree/Agree	41.5 (22)
Neutral	11.3 (6)
Disagree/Strongly disagree	1.9 (1)
**Nurses providing telenursing interventions should receive targeted training and mentoring**	
Strongly agree/Agree	88.7 (47)
Neutral	9.4 (5)
Disagree/Strongly disagree	1.9 (1)

**Table 4:** Nurses’ barriers to telenursing.

Nurses’ Barriers	% (N)
**Do you think the use of telenursing could improve communication within the care team and patient safety?**	
Both disagree	3.8 (2)
Neither agree nor disagree on both aspects	9.4 (5)
Both agree	73.6 (39)
Agree with the communication	9.4 (5)
Agree with the patient safety	3.8 (2)
**The use of telenursing enhances the empathic nurse-patient relationship**	
Strongly agree/Agree	69.8 (37)
Neutral	18.9 (10)
Disagree/Strongly disagree	11.3 (6)
**Nurses’ barriers to using telenursing include lack of familiarity with digital devices**	
Strongly agree/Agree	83 (44)
Neutral	11.3 (6)
Disagree/Strongly disagree	5.7 (3)
**Nurses’ barriers to telenursing use are cultural**	
Strongly agree/Agree	79.3 (42)
Neutral	13.2 (7)
Disagree/Strongly disagree	7.5 (4)
**Nurses’ barriers to using telenursing are related to lack of support and involvement from management**	
Strongly agree/Agree	92.5 (49)
Neutral	7.5 (4)
Disagree/Strongly disagree	0 (0)
**Involving nurses in organisational innovation goals could increase their utilisation and satisfaction**	
Strongly agree/Agree	88.7 (47)
Neutral	7.5 (4)
Disagree/Strongly disagree	3.8 (2)
**Nurses’ barriers to using telenursing are related to lack of time**	
Strongly agree/Agree	71.7 (38)
Neutral	17 (9)
Disagree/Strongly disagree	12.4 (6)

**Table 5:** Patient barriers and area of use.

Patients’ Barriers	% (N)
**Patient barriers to telenursing include lack of digital literacy**	
Strongly agree/Agree	88.7 (47)
Neutral	5.7 (3)
Disagree/Strongly disagree	5.7 (3)
**Patients’ barriers to telenursing are cultural**	
Strongly agree/Agree	67.9 (36)
Neutral	24.5 (13)
Disagree/Strongly disagree	7.6 (4)
**Patient/caregiver information and education interventions could reduce patient barriers**	
Strongly agree/Agree	90.6 (48)
Neutral	9.4 (5)
Disagree/Strongly disagree	0 (0)
**Overall, patients may be in favour of telenursing**	
Strongly agree/Agree	75.5 (40)
Neutral	9.4 (5)
Disagree/Strongly disagree	0 (0)
**Area of use**	**% (N)**
**In the post-acute phase, telenursing is effective in monitoring vital signs, signs and symptoms**	
Strongly agree/Agree	84.9 (45)
Neutral	9.4 (5)
Disagree/Strongly disagree	5.7 (3)
**In the acute phase of the disease, telenursing is effective in providing support**	
Strongly agree/Agree	58.4 (31)
Neutral	13.2 (7)
Disagree/Strongly disagree	28.3 (15)
**Telenursing is effective in supporting chronic conditions**	
Strongly agree/Agree	92.4 (49)
Neutral	7.5 (4)
Disagree/Strongly disagree	0 (0)
**Telenursing is effective in palliative care**	

**Table 6:** Correlation and Regression Findings.

Dependent Variable (Y)	Independent Variable (X)	β (Coefficient)	R^2^	F	*p*-Value
C4—Communication	Gender	NA	NA	5.515	0.023
C7—Telenursing Guidelines	Gender	NA	NA	4.701	0.035
C3—Time savings	C2—Level of knowledge	0.651	0.424	36.12	<0.001
C4—Communication	C2—Level of knowledge	0.474	0.225	14.73	<0.001
C5—Professional liability	C2—Level of knowledge	0.591	0.349	27.84	<0.001
C6—Issues with professional liability	C2—Level of knowledge	0.373	0.139	8.2	0.006
C8—Useability	C2—Level of knowledge	0.605	0.366	29.39	<0.001
C11—Professional growth	C2—Level of knowledge	0.315	0.099	5.53	0.021
C9—Favourite tool	D1—Improved communication	0.375	0.141	8.5	0.006
D5—Organisational barriers	C3—Time savings	0.449	0.201	11.89	0.001
D6—Management support	C3—Time savings	0.331	0.11	6.3	0.015
D10—Patient barriers	C4—Communication	0.49	0.24	15.98	<0.001

β = unstandardised regression coefficient; R^2^ = proportion of variance in the dependent variable explained by the model; F-statistic for the significance test of the entire regression model; NA = Not Applicable.

## Data Availability

Due to privacy or ethical restrictions, the datasets generated or analyzed during this study are available from the corresponding author on reasonable request.

## Supplementary Materials

The following supporting information can be downloaded at: https://www.mdpi.com/article/10.3390/healthcare14162491/s1, Supplementary File S1: Questionnaire; Supplementary File S2: STROBE checklist.

## Abbreviations

The following abbreviations are used in this manuscript:

STROBE	Strengthening the Reporting of Observational Studies in Epidemiology
KAB	Knowledge, Attitudes, Behaviours
PhD	Doctor of Philosophy
MSN	Master of Science in Nursing
