# Supplementary material for: Acceptability and Usefulness of Telenursing from Nurses’ Perspectives: A Descriptive Pilot Study
Source: Healthcare (Basel). 2026 Aug 11;14(16):2491. doi: 10.3390/healthcare14162491 (PMC13512270; doi:10.3390/healthcare14162491)
Supplement: Supplementary file 1 [file healthcare-14-02491-s001.zip › Supplementary File S1 - Questionnaire.pdf]

# PREDICTIVE FACTORS FOR THE USE OF TELENURSING AMONG NURSES: A PILOT STUDY.

*Dear Colleague,*

*'Telenursing' refers to a range of nursing strategies and services that use information and communication technologies (ICTs) when the nurse and patient (or two nurses) are not in the same place. This survey aims to investigate the factors that influence nurses' use of telenursing in clinical practice. This study will improve our understanding of this innovative nursing approach, which aims to enhance current clinical practice, clinical outcomes and nurse and patient satisfaction.*

*Completion of the tool is exclusively reserved for nurses (not students or other healthcare professionals) and will take approximately 10–15 minutes. It will provide invaluable insight into this evolving field. Participation in this study is entirely voluntary, free of charge and non-mandatory, and will not result in any personal benefit or advantage. Similarly, choosing not to participate will not result in any personal risk or disadvantage.*

*To ensure the reliability of the results, please complete the questionnaire only once. The study manager will collect and process the data anonymously in his capacity as Data Controller, and no information will be traceable back to the opinions of individual participants. This study has been approved by a Regional Ethics Committee with identification number 227/IRE/24.*

*Please read the following information carefully before taking part in the study.*

Dear colleague,

We are conducting a study entitled 'Predictive factors for the use of telenursing among nurses: a Pilot Study', and we require the collaboration of nurses who meet the necessary criteria, such as yourself.

This document is intended to provide information about the nature of the study, its purpose, and what it will involve. It is important that you understand why the study is being conducted and what it involves before you decide whether to participate.

Please read this information carefully. If you have any questions, queries or requests for further clarification, please send them to XXXXXXX@XXX (Principal Investigator.). Feel free to take all the time you need.

Your participation is entirely voluntary. Refusing to participate will not result in any penalty.

If you agree to participate, you will be asked to sign the consent form below to confirm that you have read and understood the form and the objectives and purposes of the study. You will also have the option to withdraw your consent at any time without prejudice.

**WHAT IS THE AIM OF THE STUDY?**

This study aims to investigate Italian nurses' perspectives on the use of telenursing in clinical practice. Specifically, the study aims to: identify predictive factors for the use of telenursing among nurses using a tool developed by the authors. The main psychometric properties of the study will be tested and confirmed in a subsequent study involving a larger population.

## WHO IS ORGANISING/PROMOTING THIS STUDY?

The study has been conducted with the support of an Italian National Cancer Institute.

## WHAT DOES THIS STUDY INVOLVE?

All interested nurses will be offered the opportunity to participate in the study. Data will be collected via an online survey, for which the link will be distributed to participants via major social media platforms, messaging systems and email. Gathering participants' views on telenursing will help us gain a better understanding of the phenomenon under investigation, in preparation for the implementation and dissemination of telenursing services and interventions.

## WHAT WILL HAPPEN IF I DECIDE TO TAKE PART?

Participants will be asked to give consent to complete a multiple-choice questionnaire. Participation in this research project is voluntary, free of charge and anonymous. It is also non-mandatory and will not result in any personal benefit or advantage. Similarly, choosing not to participate will not result in any personal risk or disadvantage.

## HOW LONG WILL IT TAKE TO COMPLETE THE STUDY?

Completing the questionnaire should take around 10–15 minutes, and your responses will contribute significantly to achieving the study's objectives. You may leave the questionnaire at any point.

## AM I OBLIGED TO PARTICIPATE? WHAT WOULD HAPPEN IF I CHANGED MY MIND?

Participation in this study is entirely voluntary. Refusal to participate will not result in any penalty. If you agree to participate, you have the right to withdraw at any time without prejudice. However, the study coordinator may decide to terminate your participation at any time due to unforeseen circumstances, in your best interests, for administrative reasons, or for similar reasons.

If this happens, no further procedures will be carried out as part of the study and no further anonymous data relating to you will be collected, except for the possible use and processing of previously obtained anonymous data, to avoid invalidating the study's results.

f you require further clarification on the purpose of the study, how data will be collected and analysed, or how results will be disseminated, please contact the Principal Investigator, via email XXXXXX@XXXX. You can also use this address to withdraw your consent to participate in the study.

We would like to thank you in advance for your valuable contribution to this study. The authors hope that the results of this study will encourage the adoption of telenursing by nurses and patients alike, thereby improving the quality and safety of care and ultimately strengthening the nurse-patient relationship, which remains at the heart of nursing care, even when delivered remotely.

☐ ***I consent to participating in the study.***

☐ ***I do not consent to participating in the study.***

If you decline, you will not be able to proceed with the questionnaire.

Thank you.

## FORM for WITHDRAWING consent to participate in the study
## Please send it by email to XXXXX@XXXX.

I, the undersigned, declare that I wish to withdraw my consent to participate in the study, given on __________, and I am aware that this will have no repercussions for me.

Signature______________________________________________________________Date________

**The person responsible for collecting the revocation is the contact person for the study.**

Name, surname and signature_______________________________________________Data________

### SECTION A: PERSONAL INFORMATION

**A1. Gender:**

☐ Male

☐ Female

☐ Prefer not to say

☐ Other ______

**A2. Nationality:**

☐ Italian

☐ Other _____________

**A3. Region of origin:**

☐ Northern Italy

☐ Central Italy

☐ Southern Italy

☐ Islands

**A4. Age (in full years):**

☐ 20-25

☐ 26-30

☐ 31-35

☐ 36-40

☐ 41-45

☐ 46-50

☐ 51-55

☐ 56-60

☐ >60

## SECTION B: PROFESSIONAL ACTIVITY

**B1. Year of obtaining your nursing qualification.:**

_________________________

**B2. Nursing qualification:**

☐ Laurea in Infermieristica (Bachelor's Degree in Nursing)

☐ Diploma di laurea (University Diploma – pre-Bologna system)

☐ Diploma regionale o equipollente (Regional or equivalent nursing diploma)

**B3. Post-basic educational experiences (please indicate only the highest academic degree):**

☐ None

☐ 1st Level University Master's Degree (equivalent to postgraduate diploma)

☐ Master’s Degree (Laurea Magistrale) (equivalent to MSc/MA)

☐ 2nd Level University Master’s Degree (post-master specialization)

☐ PhD (Doctorate of Research)

**B4. Work experience (in years):**

☐ 0-2

☐ 3-5

☐ 6-10

☐ 11-15

☐ 16-20

☐ 21-25

☐ 26-30

☐ >30

**B5. Care setting in which you are currently employed. If you are currently unemployed, please indicate the care setting in which you were employed for the longest period of time:**

☐ Public Hospitale

☐ Private Hospital

☐ Nursing Home

☐ Local Clinic

☐ Home care

☐ Hospice

☐ Freelance

☐ Emergency

☐ Seeking first employment

☐ Retired

☐ Other ________

**B6. Current clinical context:**

☐ Medicine

☐ Surgery

☐ Paediatrics

☐ Critical care/emergency

☐ Outpatient clinic/day hospital

☐ Primary Care

☐ Education

☐ Research

☐ Management

☐ Other ___________

**B7. Work location:**

☐ Northern Italy

☐ Central Italy

☐ Southern Italy

☐ Islands

**SECTION C: KNOWLEDGE AND ATTITUDES TOWARDS THE USE OF TELENURSING**

*Rate your level of agreement with the following statements:*

**C1. I use the Internet more for work than leisure.**

☐ Strongly disagree

☐ Disagree

☐ Neutral

☐ Agree

☐ Strongly agree

**C2. My knowledge on the use of telenursing is good:**

☐ Strongly disagree

☐ Disagree

☐ Neutral

☐ Agree

☐ Strongly agree

**C3. Telenursing saves time in clinical practice:**

☐ Strongly disagree

☐ Disagree

☐ Neutral

☐ Agree

☐ Strongly agree

**C4. Remote nurse-patient interactions are facilitated by open-ended questions and attention to non-verbal language, emotional and behavioural aspects:**

☐ Strongly disagree

☐ Disagree

☐ Neutral

☐ Agree

☐ Strongly agree

**C5. Telenursing has the same professional, ethical and legal responsibilities as traditional care to provide safe, competent, compassionate and ethical care:**

☐ Strongly disagree

☐ Disagree

☐ Neutral

☐ Agree

☐ Strongly agree

**C6. Professional liability issues in the use of telenursing include technology failure, confidentiality of health information, consistency of delivery methods, and telenursing-related skills:**

☐ Strongly disagree

☐ Disagree

☐ Neutral

☐ Agree

☐ Strongly agree

**C7. Guidelines for telenursing interventions and their use to support nurses are currently not specifically recommended:**

☐ Strongly disagree

☐ Disagree

☐ Neutral

☐ Agree

☐ Strongly agree

**C8. I would feel comfortable providing telenursing interventions:**

☐ Strongly disagree

☐ Disagree

☐ Neutral

☐ Agree

☐ Strongly agree

**C9. The tool I prefer to use and consider most suitable for telenursing is (one option):**

☐ *e*-Mail

☐ Messaging

☐ Phone calls

☐ Video callas

☐ Applications an platforms

☐ Other

**C10.  I would be concerned about providing telenursing interventions:**

☐ Strongly disagree

☐ Disagree

☐ Neutral

☐ Agree

☐ Strongly agree

**C11. Telenursing enhances professional development and job satisfaction:**

☐ Strongly disagree

☐ Disagree

☐ Neutral

☐ Agree

☐ Strongly agree

**C12. Nurses providing telenursing interventions should receive targeted training and mentoring**:

☐ Strongly disagree

☐ Disagree

☐ Neutral

☐ Agree

☐ Strongly agree

**C13. Remote nursing care, in addition to traditional care, enables nurses to provide care:**

☐ Lower quality compared to traditional care

☐ Same quality as traditional care

☐ Higher quality compared to traditional care

☐ I don’t know

**C14. Did your academic training include the use of telenursing interventions?**

☐ No

☐ Yes

☐ Only eHealth

☐ Only telenursing

**C15. Do you use telenursing at work?**

☐ No

☐ Yes

**C16. Are you interested in training in the use of telenursing tools in a clinical setting??**

☐ No

☐ Yes

**SECTION D: RESISTANCE AND BARRIERS TO THE USE OF TELENURSING**

*Rate your level of agreement with the following statements:*

**D1. The use of telenursing could improve communication within the care team and patient safety:**

☐ Both disagree

☐ Neither agree nor disagree on both aspects ☐ D’accordo per ambedue gli aspetti

☐ Agree with the communication

☐ Agree with the patient safety

**D2. The use of telenursing enhances the empathic nurse-patient relationship:**

☐ Strongly disagree

☐ Disagree

☐ Neutral

☐ Agree

☐ Strongly agree

**D3. Nurses' barriers to using telenursing include lack of familiarity with digital devices:**

☐ Strongly disagree

☐ Disagree

☐ Neutral

☐ Agree

☐ Strongly agree

**D4. Nurses' barriers to telenursing use are cultural:**

☐ Strongly disagree

☐ Disagree

☐ Neutral

☐ Agree

☐ Strongly agree

**D5. Nurses' barriers to using telenursing are related to lack of support and involvement from management:**

☐ Strongly disagree

☐ Disagree

☐ Neutral

☐ Agree

☐ Strongly agree

**D6. Involving nurses in organisational innovation goals could increase their utilisation and satisfaction:**

☐ Strongly disagree

☐ Disagree

☐ Neutral

☐ Agree

☐ Strongly agree

**D7. Nurses' barriers to using telenursing are related to lack of time:**

☐ Strongly disagree

☐ Disagree

☐ Neutral

☐ Agree

☐ Strongly agree

**D8. Patients' barriers to using telenursing are related to poor digital literacy and the complexity of the technology, as well as a lack of suitable tools.:**

☐ Strongly disagree

☐ Disagree

☐ Neutral

☐ Agree

☐ Strongly agree

**D9. Patients’ barriers to using telenursing includ cultural prejudices and barriers such as mistrust of remote nursing support services and tools, or fears that remote care may replace in-person care.:**

☐ Strongly disagree

☐ Disagree

☐ Neutral

☐ Agree

☐ Strongly agree

**D10. Patient/caregiver information and education interventions could reduce patient barriers:**

☐ Strongly disagree

☐ Disagree

☐ Neutral

☐ Agree

☐ Strongly agree

**D11. Overall, patients may be in favour of telenursing:**

☐ Strongly disagree

☐ Disagree

☐ Neutral

☐ Agree

☐ Strongly agree

**D12. In the post-acute phase, telenursing is effective in monitoring vital signs, signs and symptoms:**

☐ Strongly disagree

☐ Disagree

☐ Neutral

☐ Agree

☐ Strongly agree

**D13. In the acute phase of the disease, telenursing is effective in providing support:**

☐ Strongly disagree

☐ Disagree

☐ Neutral

☐ Agree

☐ Strongly agree

**D14. Telenursing is effective in supporting chronic conditions, including cancer:**

☐ Strongly disagree

☐ Disagree

☐ Neutral

☐ Agree

☐ Strongly agree

**D15. Telenursing is effective in patient education interventions aimed at patients and caregivers:**

☐ Strongly disagree

☐ Disagree

☐ Neutral

☐ Agree

☐ Strongly agree

**D16. Telenursing is effective in palliative care:**

☐ Strongly disagree

☐ Disagree

☐ Neutral

☐ Agree

☐ Strongly agree
